# Diagnostic Yield of Comprehensive Reanalysis After Nondiagnostic Short-Read Genome Sequencing in Infants With Unexplained Epilepsy

**DOI:** 10.1212/WNL.0000000000214645

**Published:** 2026-02-13

**Authors:** Jimmy N.H. Nguyen, Maria Lachgar-Ruiz, Edward J. Higginbotham, Matthew Coleman, John Coleman, Wanqing Shao, Elizabeth Scotchman, Ashley J. Pritchard, Katrina M. Bell, Lyn S. Chitty, John Christodoulou, Paul De Fazio, Ashish R. Deshwar, Christin Eltze, Anna J.S. Griffiths, Jane Hassell, Puneet Jain, Marios Kaliakatsos, Nicole S.Y. Liang, Patrick Lombard, Christian R. Marshall, Catherine Marx, Lyndsey McRae, Sarah Mulhern, Ben Paternoster, Ana Perez Caballero, Neta Pipko, Jashanpreet Sidhu, Lacey Smith, Zornitza Stark, Brett Trost, Emma Wakeling, Susan M. White, Michael Yoong, Gene-STEPS Study Group, IPCHiP Executive Committee,, Natalie J. Chandler, J. Helen Cross, Ingrid E. Scheffer, Vann Chau, Annapurna Poduri, Katherine B. Howell, Sarah E.M. Stephenson, Amy McTague, Gregory Costain, Alissa M. D'Gama

**Affiliations:** 1 Program in Genetics and Genome Biology, The Hospital for Sick Children, Toronto, Ontario, Canada;; 2 Department of Molecular Genetics, University of Toronto, Ontario, Canada;; 3 North Thames Genomic Laboratory Hub, Great Ormond Street NHS Foundation Trust, London, United Kingdom;; 4 Division of Genome Diagnostics, Department of Paediatric Laboratory Medicine, The Hospital for Sick Children, Toronto, Ontario, Canada;; 5 The Centre for Applied Genomics, The Hospital for Sick Children, Toronto, Ontario, Canada;; 6 Murdoch Children's Research Institute, Melbourne, Australia;; 7 Department of Paediatrics, University of Melbourne, Australia;; 8 Children's Rare Disease Collaborative, Boston Children's Hospital, MA;; 9 UCL Great Ormond Street Institute of Child Health, Genetic and Genomic Medicine, London, United Kingdom;; 10 Victorian Clinical Genetics Services, Murdoch Children's Research Institute, Melbourne, Australia;; 11 Department of Paediatrics, University of Toronto, Ontario, Canada;; 12 Program in Developmental, Stem Cell & Cancer Biology, The Hospital for Sick Children, Toronto, Ontario, Canada;; 13 Division of Clinical and Metabolic Genetics, The Hospital for Sick Children, Toronto, Ontario, Canada;; 14 Department of Neurology, Great Ormond Street Hospital, London, United Kingdom;; 15 Department of Neurology, Royal Children's Hospital, Melbourne, Australia;; 16 Division of Neurology, The Hospital for Sick Children, Toronto, Ontario, Canada;; 17 Neurosciences Department, Great Ormond Street Hospital, London, United Kingdom;; 18 Epilepsy Genetics Program, Department of Neurology, Boston Children's Hospital, MA;; 19 Program in Molecular Medicine, The Hospital for Sick Children, Toronto, Ontario, Canada;; 20 North East Thames Regional Genetic Service, Great Ormond Street Hospital for Children NHS Foundation Trust, London, United Kingdom;; 21 Department of Paediatric Neurology, Royal London Hospital, London, United Kingdom;; 22 Developmental Neurosciences, UCL Great Ormond Street Institute of Child Health, London, United Kingdom;; 23 Department of Medicine, University of Melbourne, Austin Health, Heidelberg, Australia;; 24 Florey Institute of Neuroscience and Mental Health, Melbourne, Australia;; 25 Division of Newborn Medicine, Department of Pediatrics, Boston Children's Hospital, MA; and; 26 Department of Pediatrics, Harvard Medical School, Boston, MA.

## Abstract

**Background and Objectives:**

The highest incidence of epilepsy in childhood occurs in the first year of life. Infantile epilepsies are associated with substantial morbidity and mortality. Although most are presumed to have genetic etiologies, many infants with nonacquired epilepsy remain genetically unsolved after clinical genome sequencing. The yield of reanalysis after nondiagnostic genome sequencing in this population is unknown. We aimed to determine the diagnostic yield of comprehensive reanalysis after nondiagnostic genome sequencing in infants with unexplained epilepsy.

**Methods:**

This cohort study included infants with unexplained epilepsy or complex febrile seizures who were recruited from 4 pediatric referral centers from September 2021 to March 2024 and had nondiagnostic clinical rapid genome sequencing. We performed comprehensive reanalysis of genome sequencing data from infants and available biological parents using multiple bioinformatics pipelines through July 2025 and clinically confirmed reanalysis findings. The primary outcome was diagnostic yield of genome sequencing reanalysis, defined as the percentage of infants who received genetic diagnoses from reanalysis. The secondary outcome was clinical utility of reanalysis findings.

**Results:**

From an initial cohort of 312 infants with unexplained epilepsy who underwent clinical rapid genome sequencing, we performed comprehensive genome reanalysis in 176 infants with initially nondiagnostic results at a median age of 642 days, including 63 female patients (36%) and 30 (17%) with neonatal-onset seizures. The diagnostic yield of reanalysis was 5.1% (9/176, 95% CI 2.4%–9.5%), increasing the overall yield from 43.6% (136/312, 95% CI 38.0%–49.3%) to 46.5% (145/312, 95% CI 40.8%–52.2%). Of the new diagnoses, 6 involved variants not reported by clinical laboratories (2 single nucleotide variants, 2 structural variants, 1 tandem repeat expansion, 1 mosaic variant) and 3 involved previously reported variants of uncertain significance with new evidence. All diagnoses had clinical utility.

**Discussion:**

Comprehensive reanalysis after nondiagnostic rapid genome sequencing has utility for infants with unexplained epilepsy. Our findings support implementation of reanalysis within 1–2 years after nondiagnostic genomic sequencing into routine clinical care of children with unexplained epilepsy and the expansion of clinically accredited genomic sequencing to include complex and noncoding variant detection.

## Introduction

Epilepsy is the most common childhood neurologic disorder, with highest incidence in the first year of life.^1-4^ Infants with epilepsy, especially developmental and epileptic encephalopathies (DEEs), have high rates of morbidity, notably drug-resistant epilepsy and poor neurodevelopmental outcomes, and increased mortality.^1-4^ Genetic diagnosis in this population informs clinical management, including access to precision therapies, and has the potential to improve outcomes.^1-4^

Short-read genome sequencing (GS) is the most comprehensive clinical genetic test available and can detect most epilepsy-associated pathogenic variants.^5-9^ Gene-Shortening Time of Evaluation in Pediatric epilepsy Services (STEPS) is a multicenter prospective study designed to determine the utility of clinically accredited rapid GS (rGS, turnaround time within a few weeks) in infants with unexplained epilepsy.^10^ We reported a 43% diagnostic yield of rGS in our first year of enrollment; thus, most infants remained genetically unsolved even after rGS.

Comprehensive reanalysis in the research setting has the potential to detect additional genetic diagnoses through advanced analysis for additional variant types and incorporation of new information.^7,11-13^ The utility of comprehensive GS reanalysis in epilepsy remains unknown. We aimed to investigate this in our rigorously phenotyped and longitudinally followed Gene-STEPS cohort of infants with epilepsy and nondiagnostic rGS.

## Methods

### Study Design and Cohort

Gene-STEPS is the flagship project of the International Precision Child Health Partnership, a consortium of 4 tertiary pediatric centers in Australia (Murdoch Children's Research Institute and The Royal Children's Hospital), Canada (The Hospital for Sick Children), the United Kingdom (University College London Great Ormond Street Institute of Child Health), and the United States (Boston Children's Hospital).^14^ From September 2021 to March 2024, we consecutively recruited infants who presented to the 4 centers with unexplained new-onset epilepsy or complex febrile seizures (and biological parents where available). We included infants with seizure onset before 12 months of age who were enrolled within 6 weeks of site presentation and excluded infants with simple febrile seizures, acute provoked seizures, or a known acquired or genetic cause. We performed short-read rGS in clinically accredited laboratories as previously described.^10^ For infants with nondiagnostic rGS, we performed comprehensive reanalysis of GS data.

### Clinical Data

In addition to data collected from the electronic medical record at enrollment, which included demographics, epilepsy (seizure types, EEG findings) and development details, other neurologic and non-neurologic features, MRI findings, and other genetic testing,^10^ we collected additional data to assist reanalysis, including any new data in the above categories and if applicable, the age and cause of death. Epilepsy syndromes were classified based on International League Against Epilepsy criteria.^10,15^

### Reanalysis

We performed reanalysis using multiple computational workflows, which were harmonized across sites and implemented locally by each site for their GS data (details in eMethods). In brief, we first reassessed variants of uncertain significance (VUSs) on the rGS report and manually reviewed GS data for a second variant when the rGS reported a single pathogenic/likely pathogenic variant in a gene associated with a relevant autosomal recessive disease. We next performed reanalysis for single nucleotide variants (SNVs), small insertions and deletions (indels), and copy number variants (CNVs) (eFigure 1A), using a consensus epilepsy gene list we developed (n = 1,112 genes, available on PanelApp Australia^16,17^ [panel version 1.267]), followed by genome-wide analysis. We then performed analysis for variant types not routinely assessed by clinical GS, as feasible at each site (details in eMethods), including structural variants (SVs), short tandem repeat (STR) expansions, mobile element insertions (MEIs), mosaic variants, mitochondrial variants, and noncoding variants (focusing on regulatory elements and “poison” exons)^18^ (eFigure 1B). RNA analysis and orthogonal variant confirmation were performed as needed (details in eMethods).

### Variant Interpretation

Variants identified by reanalysis were classified using the same standardized criteria applied to those previously detected by rGS.^10,19-22^ Pathogenic/likely pathogenic variants in genes with consistent phenotypes and modes of inheritance were considered diagnostic. Selected VUSs were considered clinically diagnostic by expert consensus when supporting evidence, including genotype-phenotype match and additional investigations, favored pathogenicity and no evidence ruled out pathogenicity.^10^ Variant interpretation was discussed at multisite meetings, and variant classification was confirmed by clinically accredited laboratories at each site following standardized criteria (American College of Medical Genetics and Genomics or Association for Clinical Genomic Science).^10,19-22^

### Clinical Confirmation and Utility

To allow return of reanalysis results to families and clinicians, reclassification and clinical confirmation were performed by clinically accredited laboratories where available. Clinical utility of reanalysis findings was assessed as previously described.^10^

### Statistical Analysis

We analyzed summary statistics for demographic, clinical, and genetic features. Diagnostic yield was defined as the percentage of cases in which pathogenic/likely pathogenic variants or VUSs considered clinically diagnostic were identified by reanalysis.

### Standard Protocol Approvals, Registrations, and Patient Consents

The Gene-STEPS study was approved by the institutional review boards of Melbourne Children's Campus (Murdoch Children's Research Institute and The Royal Children's Hospital) in Australia; The Hospital for Sick Children in Canada; University College London Great Ormond Street Institute of Child Health in the United Kingdom; and Boston Children's Hospital in the United States. Parents provided written informed consent. Data are reported per Strengthening the Reporting of Observational Studies in Epidemiology guidelines.

### Data Availability

Deidentified participant demographic and clinical data will be made available per site-specific regulations with data use agreements on request to the corresponding author. Reported variants were deposited into public databases (e.g., ClinVar) per the policies of the clinically accredited laboratories that performed the genome sequencing and clinical confirmation.

## Results

### Study Cohort

From September 1, 2021, to March 31, 2024, 312 infants with unexplained seizures were enrolled and underwent rGS (a subset have been previously reported),^10^ with genetic diagnoses identified in 136 (43.6%). Thus, 176 infants with presumed genetic epilepsy remained genetically unsolved after rGS, including 63 female patients (36%) and 30 (17%) with neonatal-onset seizures (Table 1). The median time from the clinical rGS report to research reanalysis was 468 days (interquartile range [IQR] 312–604 days), and the median age at reanalysis was 642 days (IQR 434–820 days).

**Table 1:** Cohort Characteristics

	Total (n = 176)
Demographics	
Sex reported at birth, n (%)	
Male	113 (64)
Female	63 (36)
Parent-reported race/ethnicity, n (%)	
Asian	38 (22)
Black	12 (7)
Middle Eastern	8 (4)
White	86 (49)
Multiple	9 (5)
Other	23 (13)
Clinical features	
Seizure onset, n (%)	
Neonatal (<44 wk postmenstrual age)	30 (17)
Infantile	146 (83)
Epilepsy syndrome at seizure onset, n (%)	
DEEs	97 (55)
Self-limited epilepsies	15 (9)
Other	64 (36)
Development (at time of reanalysis), n (%)^a^	
Developmental delay	89 (52)
Normal development	81 (48)
Deceased (at time of reanalysis), n (%)	
Deceased	6 (3)
Living	170 (97)
MCD, n (%)	
Yes	28 (16)
No	141 (80)
Unclear/unknown	7 (4)
Type of rGS, n (%)	
Trio	163 (92)
Duo	10 (6)
Singleton	3 (2)

Abbreviations: DEE = developmental and epileptic encephalopathy; MCD = malformation of cortical development; rGS = rapid genome sequencing.

a Developmental delay was assessed based on electronic medical records. Six participants were excluded because of deceased status at the time of reanalysis (n = 170).

### Diagnostic Yield of GS Reanalysis

**Figure 1:**
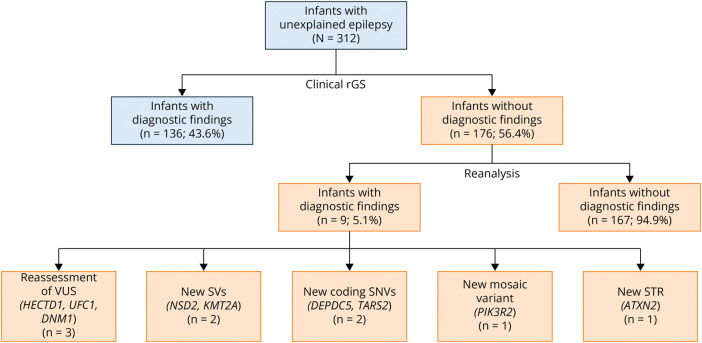
Gene-STEPS Reanalysis Overview The Gene-STEPS study recruited 312 infants with unexplained seizures who underwent clinical rGS. Among the 176 infants with nondiagnostic rGS, reanalysis identified 9 diagnostic genetic findings (shown in orange boxes). STEPS = Shortening Time of Evaluation in Pediatric epilepsy Services; rGS = rapid genome sequencing.

**Figure 2:**
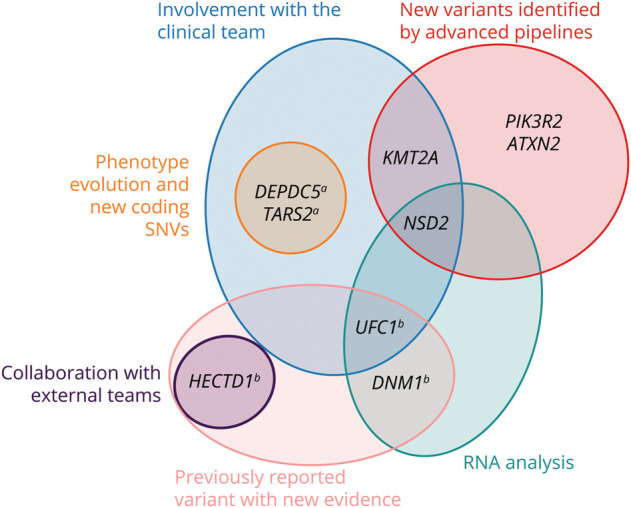
Factors Contributing to Reanalysis Diagnostic Findings Venn diagram illustrating the key factors contributing to diagnostic findings. ^a^Genes with variants of uncertain significance that were considered clinically diagnostic. ^b^Genes with variants initially reported as uncertain significance on the rGS report that were reclassified as likely pathogenic on reanalysis. rGS = rapid genome sequencing.

Comprehensive GS reanalysis identified pathogenic/likely pathogenic variants in 7 infants and VUSs considered clinically diagnostic in 2 infants (Table 2, eTable 1). The diagnostic yield of reanalysis was 5.1% (9/176; binomial 95% CI 2.4%–9.5%), bringing the diagnostic yield of the overall cohort to 46.5% (145/312; 95% CI 40.8%–52.2%) (Figures 1 and 2). Of the 10 variants identified by reanalysis in these 9 infants, 5 (50%) were coding SNVs/indels, 2 (20%) CNVs/SVs, 2 (20%) noncoding SNVs, and 1 (10%) an STR expansion. The most common mode of inheritance was de novo autosomal dominant (n = 5), followed by inherited autosomal dominant (n = 2; 1 of 2 parents affected) and autosomal recessive (n = 2). For these 9 infants, the median time from the clinical rGS report to diagnostic research reanalysis finding was 351 days (IQR 70–479).

**Table 2:** Reanalysis Findings

ID	Sex	Age at seizure onset (d)	Time from initial analysis to reanalysis (d)	Epilepsy syndrome at onset, current status	Other clinical features	Gene	Variant, zygosity, inheritance	Classification	Variant on clinical rGS report	Analyses supporting new finding or reclassification
R01	M	1	333	Other focal^a^, NA (proband deceased)	Nonepileptic myoclonus, failure to thrive	*UFC1*	NM_016406.4:c.192-6T>C (p.?), Hom, inherited	LP^b^	Yes	New evidence, clinical involvement
R02	M	115	444	DEE, drug-resistant seizures	GDD, hypotonia	*DNM1*	NM_001288739.2:c.1197-41G>A (p.?), Het, de novo	LP^b^	Yes	New evidence
R03	M	3	479	Other focal, seizure-free on medication	Central apnea, hypotonia (resolved), oropharyngeal dysphagia, NYX-related congenital stationary night blindness	*HECTD1*	NM_015382.4:c.3350T>C (p.Ile1117Thr), Het, de novo	LP^b^	Yes	New evidence
R04	F	90	558	IESS^a^, drug-resistant seizures	GDD^c^, dystonia^c^, brain atrophy^c^	*TARS2*	NM_025150.5:c.[470C>T];[1487C>T] (p.[Thr157Ile];[Thr496Ile]), Cmpd het, inherited^d^	VUS; VUS^e^	No	New evidence, phenotype evolution, clinical involvement
R05	M	180	351	IESS, drug-resistant seizures	GDD, hypotonia, microcephaly, esotropia, dolichocephaly^c^, dystonia^c^, oromotor apraxia^c^	*ATXN2*	GRCh38:chr12:g.111598950_111599019CTG[>100], Het, inherited^f^	P	No	STR assessment
R06	M	240	56	IESS^g^, drug-resistant seizures	GDD, hypotonia, macrocephaly, polymicrogyria, ventriculomegaly, TAPVC	*PIK3R2*	NM_005027.4:c.1117G>A (p.Gly373Arg), mosaic (14% VAF), de novo	P	No	Mosaic variant assessment
R07	F	122	14	Other focal, seizure-free on medication	GDD, hypotonia, dysmorphic features, failure to thrive, feeding difficulties, hirsutism	*KMT2A*	GRCh38:chr11:g.118484599_120837876inv, Het, de novo	P	No	SV assessment, clinical involvement
R08	M	127	545	Other focal, seizure-free after FCD resection	FCD, GDD^c^, hypotonia^c^	*DEPDC5*	NM_001242896.3:c.2118G>C (p.Arg706Ser), Het, inherited	VUS^e^	No	Phenotype evolution, new evidence, clinical involvement
R09	M	255	70	IESS; seizure-free off medication	GDD, hypotonia, microcephaly, dysmorphic features, IUGR, SGA, feeding difficulties	*NSD2*	GRCh38:chr4:g.1934592_1935602del, Het, de novo	P	No	SV assessment, clinical involvement

Abbreviations: Cmpd Het = compound heterozygous; DEE = developmental and epileptic encephalopathy; EIDEE = early-infantile developmental and epileptic encephalopathy; F = female; FCD = focal cortical dysplasia; GDD = global developmental delay; Het = heterozygous; Hom = homozygous; IESS = infantile epileptic spasms syndrome; IUGR = intrauterine growth restriction; LP = likely pathogenic; M = male; NA = not applicable; P = pathogenic; SGA = small for gestational age; STR = short tandem repeat; SV = structural variant; TAPVC = total anomalous pulmonary venous connection; VAF = variant allele fraction; VUS = variant of uncertain significance.

a Epilepsy syndrome classified to EIDEE at the time of reanalysis.

b Variant reclassified from VUS to LP.

c New clinical features at the time of reanalysis.

d Inherited from 1 parent each (i.e., the variants are in trans).

e Variant(s) considered clinically diagnostic.

f Parental allele size: 41 repeats.

g Now also has focal seizures.

### Previously Reported Variants

Of the new diagnoses, 3 of 9 (33%) involved reassessment of VUSs on the rGS report initially considered nondiagnostic.

Participant R01 was a male infant born to consanguineous parents who presented with early infantile DEE at birth and died at 9 days of age. rGS identified a homozygous splice region VUS in *UFC1* (c.192-6T>C). RNA sequencing from blood demonstrated a “leaky” splice site leading to exon 2 skipping in 113 of 334 reads (33.8%) (eFigure 2A), predicted to result in a frameshift and premature stop codon. Pathogenicity was further supported by segregation studies that identified the homozygous variant in an extended family member with a severe neurodevelopmental phenotype (eFigure 2B). The phenotype, segregation data, and hypomorphic variant impact (consistent with previous reports of *UFC1*-related disorder)^23^ resulted in variant reclassification to likely pathogenic.

For participant R02, a male infant who presented with an unclassified DEE with generalized and focal seizures, hypotonia, and global developmental delay (GDD), rGS identified a de novo heterozygous noncoding VUS in intron 10 of *DNM1* (NM_004408.4:c.1335+1605G>A). The variant is 5′ of exon 10a, which is encoded in 2 alternative transcripts highly expressed in the brain (NM_001288737.2 and NM_001288739.2:c.1197-41G>A, eFigure 3A). This variant is predicted to create a cryptic splice acceptor site with a higher SpliceAI^24^ score than the canonical site, leading to partial intron retention of 13 amino acids in exon 10a. A nearby recurrent splice region variant (NM_001288739.2:c.1197-8G>A) in *DNM1* has been described in individuals with DEE and similar phenotypes.^25,26^ Sanger sequencing of cDNA generated from participant-derived fibroblast cells confirmed aberrant splicing, and the variant was reclassified to likely pathogenic (eFigure 3B).

For participant R03, a male infant who presented with neonatal-onset focal epilepsy, hypotonia, central apnea, and oropharyngeal dysphagia, rGS identified a de novo heterozygous missense VUS in *HECTD1* (c.3350T>C). Although the variant was predicted deleterious, *HECTD1*, which encodes an ubiquitin ligase involved in neural tube development, was a gene of uncertain significance at the time of the initial report. Through GeneMatcher collaboration,^27^ additional individuals with *HECTD1* variants and overlapping phenotypes were identified, including 6 with epilepsy.^28^ Functional studies in mice and *C. elegans* further supported *HECTD1* as a novel neurodevelopmental disorder gene,^28^ and the variant was reclassified to likely pathogenic.

### Newly Identified Variants

For 6 of 9 diagnoses (67%), we identified new variants, including 4 cases in which reanalysis identified variant types not routinely assessed by clinical GS.

For participant R04, a female infant who presented with infantile epileptic spasms syndrome (IESS), rGS identified a heterozygous pathogenic variant in *SLC6A5*. Given that *SLC6A5*-related disorders are inherited in an autosomal recessive manner and a second pathogenic variant was not identified, this result was considered nondiagnostic. Her phenotype evolved to include oropharyngeal dysphagia, severe GDD, tonic seizures, dystonia, and brain atrophy—clinical features not consistent with an *SLC6A5*-related disorder. Reanalysis identified a compound heterozygous missense VUS in *TARS2* (c.[470C>T];[1487C>T]). *TARS2* is associated with a mitochondrial disorder of variable severity that includes epilepsy, developmental delay, and brain atrophy.^29,30^ Although both variants were detected during the initial rGS analysis, they were not reported because both were novel variants and there was limited phenotype match. By the time of reanalysis, the c.1487C>T variant had been reported in an individual with autosomal recessive *TARS2* deficiency.^29^ Participant-derived fibroblast samples showed an increased lactate-to-pyruvate ratio and reduced complex IV enzyme, suggestive of a mitochondrial disorder. Thus, we considered these variants clinically diagnostic.

**Figure 3:**
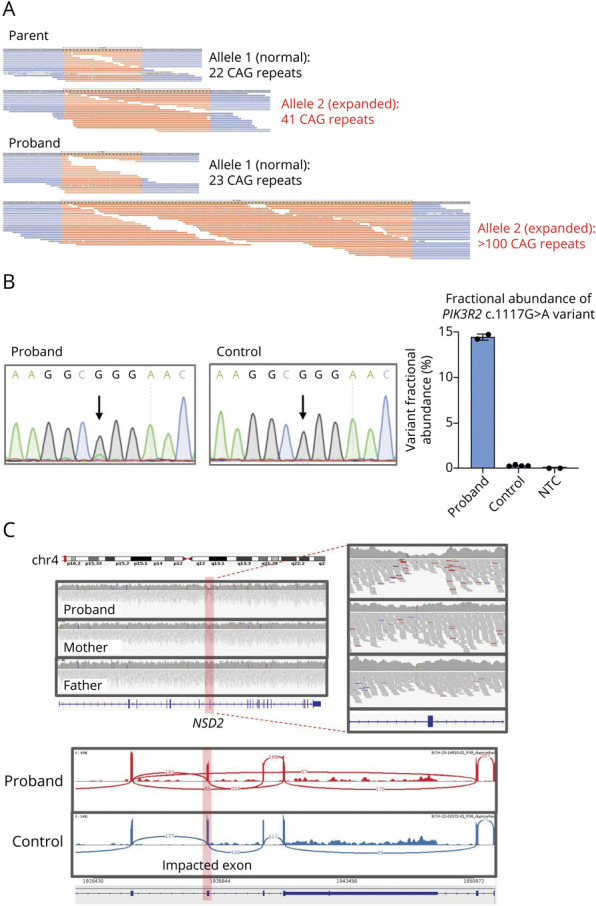
Examples of Newly Identified Variants Through Reanalysis (A) Visualization of *ATXN2* alleles for participant R05 using REViewer.^33^ Orange represents sequencing reads aligning to the CAG short tandem repeat while blue represents sequencing reads aligning to the unique flanking sequence. (B) *PIK3R2* mosaic variant confirmation in participant R06. Sanger sequencing results (left) from the proband and control. Droplet digital PCR analysis (right) quantifying the mutant allele in proband and control. Technical replicates: n = 2 (proband), n = 4 (control), n = 2 (NTC: nontemplate control). (C) Integrative Genomics Viewer (IGV) visualization (top) displaying a de novo deletion in *NSD2* overlapping exon 7 in participant R09. RNA sequencing data (bottom) confirming that the deletion creates an out-of-frame exon-skipping event.

For participant R05, a male infant with IESS and eventual focal seizures; microcephaly with cerebral, cerebellar, and vermian atrophy and delayed myelination; GDD; hypotonia; and dystonia, reanalysis identified a pathogenic STR expansion of >100 CAG repeats in exon 1 of *ATXN2* (Figure 3A), confirmed by repeat-primed PCR. Expansions of ≥35 CAG repeats in *ATXN2* lead to the neurodegenerative disorder spinocerebellar ataxia type 2 (SCA2), which typically manifests in adulthood. However, large expansions of >100 repeats have been reported in infantile-onset SCA2 with infantile seizures.^31,32^ The family had no history of SCA2, but 1 parent had a 41-repeat CAG expansion and was subsequently identified to have early symptoms of adult-onset SCA2, consistent with genetic anticipation.

For participant R06, a male infant with IESS, GDD, polymicrogyria, macrocephaly, hydrocephalus, hypotonia, and congenital heart disease, reanalysis identified a mosaic pathogenic de novo missense variant in *PIK3R2* (c.1117G>A), confirmed using Sanger sequencing and droplet digital PCR at 14% variant allele fraction (VAF; Figure 3B). The relatively low VAF accounts for lack of detection using standard clinical analysis algorithms that required a minimum VAF of 15%.

Participant R07, a female infant with focal epilepsy, GDD, hypotonia, and dysmorphic features including hirsutism and long eyelashes, was suspected clinically to have Wiedemann-Steiner syndrome, associated with pathogenic variants in *KMT2A*. Guided by this, reanalysis detected a pathogenic de novo 2.35 Mb inversion on chromosome 11q23.3, with proximal breakpoint in intron 9 of *KMT2A* and distal breakpoint in intron 8 of *GRIK4* (eFigure 4).

For participant R08, a male infant with focal epilepsy, GDD, hypotonia, and focal cortical dysplasia (FCD), rGS identified a likely pathogenic variant in *SCN1A* (c.256A>C), inherited from his unaffected mother. However, his phenotype was not typical for *SCN1A*-related epilepsies, as evidenced by seizure reduction with sodium channel blockers, lack of fever as a seizure trigger, and seizure onset, semiology, and EEGs congruent with FCD; thus, this variant was considered nondiagnostic. The FCD was resected and histopathologically confirmed as FCD IIa. He has been seizure-free and making developmental progress since resection. Reanalysis identified a missense VUS in *DEPDC5* (c.2118G>C) inherited from his unaffected father, which was detected on the initial rGS analysis but not reported because the *SCN1A* variant was considered a better explanation by the clinical laboratory. *DEPDC5* is associated with FCD II and focal epilepsy, with known incomplete penetrance,^34^ and we considered the *DEPDC5* variant clinically diagnostic.

For participant R09, a male infant with IESS, GDD, hypotonia, microcephaly, dysmorphic features, prenatal and postnatal growth restriction, and feeding difficulties, rGS identified a hemizygous VUS in *TAF1* and reanalysis identified a pathogenic de novo 1.01 kb deletion overlapping exon 7 of *NSD2*. RNA sequencing did not show a splicing impact for the *TAF1* variant and confirmed the *NSD2* deletion (Figure 3C). This small deletion was not detected by the CNV callers used by the clinical laboratory but was identified by SV-based callers on reanalysis.

### Clinical Utility

All reanalysis diagnoses had clinical utility (Table 3), including informing treatment (n = 1), prognosis (n = 8), goals of care (n = 1), new evaluation (n = 4), avoidance of unnecessary evaluation (n = 1), potential precision therapies (n = 3; *DNM1*, *ATXN2*, *DEPDC5*), and genetic counseling beyond informing recurrence risk (e.g., parental clinical management [n = 2]). For example, for participant R05, the SCA2 diagnosis explained emerging symptoms (autonomic instability), enabled avoidance of a repeat brain MRI, informed ongoing goals-of-care discussions, and stimulated additional participation in research related to antisense oligonucleotide therapy^35^; his parent also received a new SCA2 diagnosis, with immediate clinical management implications.

**Table 3:** Clinical Utility of Reanalysis Findings

ID	Gene	Any utility	Influence treatment	New workup	Avoid workup	Inform prognosis	Inform goals of care	Inform genetic counseling^a^	Potential precision therapy
R01	*UFC1*	Yes	—	—	—	—	—	Yes	—
R02	*DNM1*	Yes	—	—	—	Yes	—	—	Yes
R03	*HECTD1*	Yes	—	—	—	Yes	—	—	—
R04	*TARS2*	Yes	—	Yes	—	Yes	—	—	—
R05	*ATXN2*	Yes	Yes	Yes	Yes	Yes	Yes	Yes	Yes
R06	*PIK3R2*	Yes	—	—	—	Yes	—	—	—
R07	*KMT2A*	Yes	—	Yes	—	Yes	—	—	—
R08	*DEPDC5*	Yes	—	—	—	Yes	—	—	Yes
R09	*NSD2*	Yes	—	Yes	—	Yes	—	—	—

a As previously described,^10^ Yes in this column refers to new health implication for parents or referral of additional family members for genetic testing for the variant(s).

## Discussion

Comprehensive reanalysis after nondiagnostic rGS in a rigorously phenotyped and longitudinally followed infantile epilepsy cohort identified genetic diagnoses in 9 of 176 infants, representing a reanalysis diagnostic yield of 5.1%. The diagnoses included multiple variant types that clinically accredited laboratories face challenges in detecting and reporting. Research reanalysis allowed us to expand the variant types detected and consider phenotype evolution and new variant/gene-disease associations.

Several key factors contributed to our reanalysis diagnoses. First, we used optimized bioinformatics pipelines to detect variant types that are not routinely implemented in clinically accredited laboratories, which established 4 of 9 (44%) of our diagnoses: 2 SVs, a mosaic variant, and a STR expansion. For example, we used ExpansionHunter^33,36^ to perform STR analysis of approximately 60 disease loci and identified a rare infantile case of SCA2 with an *ATXN2* repeat expansion. Repeat expansion disorders are clinically and genetically heterogeneous, and while many are traditionally associated with adult-onset conditions, the phenotypic spectrum of some has recently expanded to infantile-onset DEEs.^37,38^ However, most clinically accredited laboratories are unable to perform STR analysis on GS data or restrict analysis to a few loci.

Second, we used RNA analysis, which is not routinely clinically available, to provide functional evidence for variant impact, which helped establish 3 of 9 (33%) of our diagnoses. In 2 cases, RNA analysis confirmed the effect of splice site variants: one resulting in a leaky splice site with exon skipping and the other leading to partial intron retention. In a third case, it confirmed the transcriptional impact of a small deletion.

Third, we had close collaboration between clinical and research teams, which helped establish 5 of 9 (56%) of our diagnoses. Gene-STEPS performs clinical rGS in infants with seizure onset before 12 months of age, which is important to rapidly identify genetic diagnoses that often affect management.^10^ However, infants may not manifest all associated features of an underlying genetic condition, and/or it may be difficult to ascertain certain features. Longitudinal phenotyping in collaboration with clinical teams and integration with reanalysis is thus important, which was made possible by transfer of the clinical rGS data to the research teams. Not included in our diagnostic count in this study is a patient who received a genetic diagnosis from initial clinical rGS and then a second genetic diagnosis through reanalysis after manifesting a new neurodevelopmental phenotype. Ongoing genetic counseling is also key because initial nondiagnostic results are not equivalent to a nongenetic etiology, and reanalysis results may have management implications for the infant and parents.

Finally, we also benefited from external collaboration, as have other GS reanalysis cohorts, which helped demonstrate a new disease-gene association and establish one of our new diagnoses.

Our 5.1% reanalysis yield is comparable to that in other GS reanalysis studies in rare disease cohorts (e.g., median 5%, IQR 3%–11% in cited studies).^11,39-46^ Variability may reflect differences in cohort composition, scope of testing, and the time interval between initial analysis and reanalysis. In our infantile epilepsy cohort, the median time to reanalysis was only 468 days and the shortest times to reanalysis diagnoses were for new variant types. It is important to note that all diagnoses identified through GS reanalysis had clinical utility and resulted in improved patient care. As new epilepsy genes have recently been identified at an average rate of 23 genes per 6 months,^47^ we anticipate that ongoing reanalyses will further increase diagnostic yield. Our findings suggest implementing reanalysis for this population within 1–2 years after nondiagnostic testing. If reanalysis resources are limited, we suggest prioritizing individuals with severe phenotypes (e.g., drug-resistant epilepsy) and/or phenotype evolution.

We acknowledge that implementing all components of our multifaceted reanalysis strategy may be challenging in many clinical laboratory settings. Based on our findings, we recommend prioritizing the following approaches into clinical laboratory workflows: First, expanding the range of variant types analyzed by incorporating additional bioinformatics pipelines has relatively high diagnostic utility—accounting for 4 of our reanalysis diagnoses—and may represent a feasible starting point for clinical laboratories. The structural and mosaic variants identified by our reanalysis were all identified within a short time interval from the initial rGS (<70 days), and although the STR expansion was identified after a longer time interval (351 days), this delay reflected the time required for data transfer to the research team rather than analytic constraint. Second, RNA analysis demonstrated high diagnostic utility when selectively applied to candidate variants predicted to affect the RNA transcript, consistent with previous reports.^48^ Third, while automation was not implemented in this study, future efforts to automate variant reanalysis may further increase diagnostic yield and efficiency in reanalysis workflows.^12^ In particular, automation may be useful for identifying variants in newly established human disease genes or variants in known human disease genes with new evidence for pathogenicity based on information in publicly available databases, as well as transferring new phenotype data (e.g., new Human Phenotype Ontology terms or International Classification of Diseases codes) from electronic medical records to clinically accredited laboratories.

Our study has several limitations. First, some complex variants are difficult to identify in short-read GS even with optimized bioinformatics pipelines and may require emerging long-read sequencing to resolve.^49,50^ We anticipate an added diagnostic yield from long-read sequencing,^49,50^ although this yield may be disease-dependent. Second, some of the cases that remain genetically unsolved may have mosaic variants at a VAF too low to be detected in standard-depth GS from blood, or that are brain-limited and not possible to detect from blood, especially those with malformations of cortical development.^51^ Third, we did not identify any cases with poison exon, mitochondrial, or MEI variants, all of which have been associated with epilepsy, likely because of our cohort size.^3,5^ Future research should investigate the added yield of long-read and deep sequencing, the contribution of additional variant types in larger cohorts, and the incorporation of automated pipelines in reanalysis workflows, and consider additional etiologic models using methods such as polygenic risk scores. In addition, studies of cost-effectiveness will be critical to implementing routine reanalysis into clinical care.

Our study demonstrates that comprehensive reanalysis of GS data improves diagnostic yield in infants with epilepsy and nondiagnostic clinical GS. By integrating advanced variant calling approaches, RNA analysis, phenotype evolution, and novel disease-gene associations, we identified genetic diagnoses in 5.1% of our cohort. We anticipate that this yield will increase as our understanding of the human genome and epilepsy pathogenesis continues to evolve and bioinformatics pipelines improve. A key strength of our study was the incorporation of a collaborative clinical and research feedback loop, an aspect that bridges the gap between research-driven discoveries and clinical practice. Clinically accredited laboratories face barriers to implementing routine reanalysis, including access to up-to-date clinical information and limited funding.^13^ Our findings highlight the importance of overcoming these barriers^13^ to implementing regular GS reanalysis in clinical settings for infants with epilepsy and individuals with rare diseases more broadly.

## Appendix Coinvestigators

**Table:**

Coinvestigators are listed at Neurology.org/N.

## Glossary

CNV: copy number variant
DEE: developmental and epileptic encephalopathy
FCD: focal cortical dysplasia
GDD: global developmental delay
GS: genome sequencing
IESS: infantile epileptic spasms syndrome
IQR: interquartile range
MEI: mobile element insertion
rGS: rapid GS
SCA2: spinocerebellar ataxia type 2
SNV: single nucleotide variant
STEPS: Shortening Time of Evaluation in Pediatric epilepsy Services
STR: short tandem repeat
SV: structural variant
VAF: variant allele fraction
VUS: variant of uncertain significance
